# The Prevalence and Correlation of Suicidal Ideation Among Nurses in King Saud University Medical City

**DOI:** 10.7759/cureus.44859

**Published:** 2023-09-07

**Authors:** Khaleel I Alyahya, Rand M Alrefaei, Leen F Almadhyani, Sarah S AlQuwayz, Mona I AlOmairini, Farah A Alsayed, Yara S Alasmari

**Affiliations:** 1 Department of Anatomy, King Saud University College of Medicine, Riyadh, SAU; 2 Department of Psychiatry, King Saud University College of Medicine, Riyadh, SAU

**Keywords:** nurses, occupational health, suicide, anxiety, depression

## Abstract

Objectives

The aim of the study is to measure the prevalence of suicidal ideation among nurses at King Saud University Medical City, compare its prevalence between male and female nurses, and identify the potential risk factors.

Methods

We conducted a cross-sectional study. The questionnaire was distributed to nurses via email. It consisted of demographics, Depression, Anxiety and Stress Scale (DASS21), and Suicidal Ideation Scale (SIS). We used the Statistical Package for the Social Sciences (SPSS) statistical software for analysis.

Results

The total number of participants was 419. The estimated prevalence of suicidal ideation among nurses was 24.58%. The prevalence among female and male nurses was 24.67% and 23.68%, respectively. Moreover, we found that nurses who are non-Muslim, single, and living by themselves are highly correlated with suicidal ideation. Depression, stress, and anxiety are also significantly associated with suicidality, with depression being the most significantly related to suicidal ideation.

Conclusion

Nurses who experienced depression, anxiety, and stress had an increased likelihood of suicidal ideation. This study demonstrates the need to raise awareness of depression, anxiety, and stress in order to prevent suicidal ideation among nurses. Further research is needed to develop measures of successful monitoring and prevention.

## Introduction

Suicide is defined as death induced by self-injurious behavior with the intention of dying as a result of the behavior, whereas suicidal ideation is the act of considering, contemplating, or planning suicide [1]. Suicidal behaviors and thoughts are two of the most common reasons for emergency room admissions [2]. According to the World Health Organization (WHO), 800,000 deaths per year are caused by suicide. However, this number is likely to be greatly underestimated due to the poor accessibility and low reporting on suicide and suicide attempts across the world [3]. Suicidality rates differ widely between countries, with developed countries having the biggest burden [4], whereas in low-and-middle-income countries, it comprises 79% of global suicides. Suicide is estimated to be the 18th leading cause of death, adding up to 1.4% of deaths worldwide in 2016 [5].

The increased risk of suicide in people of different ages is correlated to sex, race, mental disorders, social stressors, recent loss, and emotional states [6]. Risk factors are important to consider; however, anyone who presents with alarming suicidal signs might be at a greater risk and need urgent assistance, regardless of their history [7]. Evidence also indicates that people who are single, divorced, and widowed are more likely to commit suicide than those who are married. As for gender, males make fewer suicide attempts but use more fatal methods in comparison to females, who are two to three times more likely to attempt suicide. Moreover, research shows that suicide is associated with occupational, financial, or legal distress, especially among the middle-aged. Agitation, insomnia, hopelessness, and acute psychiatric illnesses such as depression are the most prevalent psychological factors, whereas impulsivity and access to lethal methods are dynamic factors that all may lead to suicide [6].

Nursing is a demanding profession that takes a significant physical and mental toll on the staff [8]. Nurses are expected to be humane, compassionate, competent, and conscientious in a challenging work environment [9]. On a daily basis, nurses find themselves dealing with deteriorating patients, frequent cases of death, and family bereavement, all while having to provide professional and appropriate support to the patients and their families. [10]. We chose nurses as our sample due to the many mental health hurdles they face, which may result in low quality of care provided. For many years, nurses have been under-appreciated, have been neglected research-wise, and have inadequate suicidal prevention programs.

According to previous research, nurses have a high risk for suicide. They have been dealing with a lot of scrutiny in recent years, partly due to the increased number of populations with complex health problems, among other potential reasons [11-13]. Therefore, in light of the escalating stressors that nurses have to deal with, and the fact that fatal medications are readily accessible to them, the high risk of suicide might not be a particularly surprising result. A recent English study found that the risk of suicide was increased exclusively in female physicians and nurses, not in males [14].

Suicide can be triggered by a difficult life event, such as the loss of a partner or the loss of employment, which are forms of emotional distress [15]. In a Taiwanese study, 40% of nurses reported having occupational stress, which contributed to 10.5% of suicidal ideation [16]. With the development of medical practices, the public may expect more from nurses, placing them at higher risk of psychological illness, particularly depression, which may develop as a result of stress and burnout [17]. Psychiatric illnesses are commonly considered as a potential cause of suicide, as depression commonly coexists with those ideations and impacts them [18], and they are both recognized as major public health issues [19]. In recognition of the link between suicide and mental illness, the majority of those who took their own life did so because they were depressed, thus making suicidal ideation a significant predictor of death by suicide [20,21]. Guidelines related to nurses' support concerning suicidal ideation and attempted suicides were found to be insufficient.

In Canada, a study was conducted on 7,358 nurses from a web-based survey, which was promoted and distributed by the Canadian Federation of Nurses Unions (CFNU), and found 10.5% prevalence of suicidal ideation, 4.6% planning, and 0.7% attempts in the last year. In comparison to the results that were carried out on the basis of a lifetime duration, the prevalence of suicidal ideation was 33%, planning was 17%, and attempts were 8% [22]. A study conducted in Taiwan concluded that one-fifth of nurses have had suicidal ideation over the course of one week [16]. Also, in China, a meta-analysis compared the prevalence of suicidal ideation between nurses and the general Chinese population and found that the percentage was 10.8% and 3.9% in nurses and the general Chinese population, respectively [23]. Previous studies conducted in Australia found that female nurses have committed suicide 98% more than male nurses did [24]. In general, most studies clearly showcase the relatively high prevalence of suicidal ideation among nurses. We hypothesize that more than 18.3% of working nurses are prone to suicidal ideation [16] and that female nurses are at a higher risk in comparison with male nurses [24]. We also hypothesize that the most crucial associated risk factors of suicidal ideation in nurses include depression, anxiety, and symptoms of stress, and the prevalence of each is 35%, 37%, and 41%, respectively [25].

## Materials and methods

We carried out a cross-sectional study among nurses working at King Saud University Medical City (KSUMC). It is one of the biggest governmental research and educational medical cities in Riyadh, Saudi Arabia. The study took seven months to be completed from June 2021 to December 2021. A sample size of 276 nurses from all departments was calculated using a single proportion equation by assuming the prevalence of suicidal ideation among nurses as 18.3% with precision of 5% at alpha with 0.05. We assumed a non-response rate of 20% of the sample calculated, which was 46 nurses.

We used an anonymous, self-administered, validated questionnaire that was distributed via a Google Forms link by the nursing department to reach all the accessible nurses working at KSUMC. To further ensure that the link reaches our intended sample pool, we asked the head nurses in each department to manually distribute the link among their nursing staff. The nurses who answered the questionnaire did so voluntarily, which may have led to a selection bias. Both male and female nurses working at KSUMC were included in the study, whereas nurses working at other medical cities were excluded. Suicidal ideation was the dependent outcome variable, whereas age, gender, nationality, marital status, religion, department, years of experience, living conditions, stress, anxiety, and depression were the independent variables for this study.

The questionnaire consists of a total of 39 close-ended questions that were used for data collection, which had three main sections all written in English. The first section contained eight demographic information that included age, gender, nationality, marital status, religion, living conditions, department, and years of experience. The second section was about the Depression, Anxiety, and Stress Scale (DASS21) [26], which was used to assess the associated risk factors contributing to suicidal ideation (Appendix A). It contains 21 items that are divided into three categories (depression, anxiety, and stress). The participants answered a four-point scale regarding their mental state during the past week according to the statement asked. Each answer is assigned a score: 0 “Did not apply to me at all - never,” 1 “Applied to me to some degree, or some of the time - sometimes,” 2 “Applied to me to a considerable degree, or a good part of time - often,” and 3 “Applied to me very much, or most of the time - almost always”, with a total score ranging from 0 to 126. The final part of the questionnaire was the Suicidal Ideation Scale (SIS), which is a clinical tool consisting of 10 statements that assess a person's suicidal risk by identifying warning signs of self-injury in the past week. Responses were arranged on a five-point scale: 1 “Never,” 2 “Infrequently,” 3 “Sometimes,” 4 “Frequently,” and 5 “Always,” with a total score ranging from 10 to 50 (Appendix B). Therefore, we set a score of 11 as a cutoff for suicidal ideation. We considered the participants who scored 10 to be having no suicidal ideation [24]. At the end of the questionnaire, psychiatric care and counseling were recommended to ensure everyone’s safety.

Data were collected through Google Forms and then exported to Microsoft Excel, where the coding has been done. Then, the data were analyzed using SPSS Version 26.0 (IBM Corp., Armonk, NY). Descriptive statistics (frequencies, percentages, mean, and standard deviation) have been used to describe the categorical and quantitative variables. As for the inferential tests, one-way analysis of variance (ANOVA) and independent t-test have been used to compare the mean values of outcome variables across the categorical study variables. If the p-value is ≤0.05 with a 95% confidence interval, the test is considered significant. As for the missing data, we ensured that all data were coded correctly and there were no missing values in each variable. As for confounding factors, further studies should attempt to verify our results after considering potential confounders such as financial income or sleeping disorders which nurses may suffer from.

Prior to conducting the study, the Ethics Committee Approval was obtained from the Institutional Ethics Committee of King Saud University, Riyadh, Saudi Arabia, and a letter of consent was given with project No. E-21-6128 on August 23, 2021. Ethical guidelines were followed, and authors had no conflicts of interest.

## Results

When collecting data, we received more than the minimum required sample size, with a total of 419 responses from all nursing departments. This study on suicidal ideation among nurses of KSUMC used an online survey to collect the data and used different statistical tests. The first portion of the survey was about the demographic characteristics. According to the findings, the respondents included males (9.1%) and females (90.9%), which showed the dominance of females in the nursing profession. It also revealed that the age group of respondents was 20-29 years (11.2%), 40-49 years (24.3%), 50 years and more (12.1%), with the majority of the respondents fall in the age group of 30-39 years (52.3%). When the nationality and religion of the respondents were analyzed, it was observed that non-Saudi nurses were 94%, whereas only 6% were Saudi nationals. Similarly, non-Muslims in the survey were 85% and Muslims were 15%. It was also observed that 69.7% of the respondents were married whereas (30.3%) were single, which clearly depicted that married people were the majority. Moreover, living conditions is another important factor because it has an impact on mental health. A great portion of the sample (47%) were found to live with their families, whereas 24.8% were living with their friends and 27.9% were living alone. Moreover, the departments of KSUMC included in analysis consisted of the Emergency Medicine Department (9.5%), Gynecology Department (8.4%), Outpatient Department (9.3%), Surgery (12.6%), Pediatrics Department (7.6%), Operating Rooms (5.7%), Medicine (9.3%), Critical Care (7.2%), Ophthalmology (5.5%), Dental Nurse (11.9%), and other departments (12.9%). Similarly, years of experience in the nursing profession were as follows: less than five years of experience (13.6%), 5-10 years of experience (28.4%), 10-20 years of experience (47.3%), and more than 20 years of experience (10.7%). That means the majority of nurses had fewer years of nursing experience, ranging from 10 to 20 years (Table 1).

**Table 1:** Distribution of sociodemographic characteristics of the study subjects (N=419).

Variable		N (%)
Gender	Female	381 (90.9)
	Male	38 (9.1)
Age	20-29 years	47 (11.2)
	30-39 years	219 (52.3)
	40-49 years	102 (24.3)
	50 years and more	51 (12.1)
Nationality	Saudi	25 (6)
	Non-Saudi	394 (94)
Marital status	Single	127 (30.3)
	Married	292 (69.7)
Religion	Muslim	63 (15)
	Non-Muslim	356 (85)
Living condition	With family	198 (47.3)
	With friends	104 (24.8)
	By myself	117 (27.9)
Department	Department of Emergency Medicine	40 (9.5)
	Obstetrics and Gynecology	35 (8.4)
	Outpatient Department	39 (9.3)
	Surgery	53 (12.6)
	Pediatrics	32 (7.6)
	Nursing support services	17 (4.1)
	Operating rooms	24 (5.7)
	Medicine	39 (9.3)
	Critical care	30 (7.2)
	Ophthalmology	23 (5.5)
	Dental nurse	50 (11.9)
	Others	54 (12.9)
Years of experience	Under 1 year	9 (2.1)
	1-5 years	48 (11.5)
	5-10 years	119 (28.4)
	10-20 years	198 (47.3)
	More than 20 years	45 (10.7)

In Table 2, the suicidal score and DASS21 scores were recorded among the participants. It shows the mean and standard deviation values. The table highlights that the Suicidal Ideation Scale overall score ranged from 10 to 43, with a mean of 11 and a standard deviation of 4. The depression score ranged from 0 to 21, with a mean of 3.94 and a standard deviation of 4.28. The anxiety score ranged from 0 to 20, with a mean of 4.54 and a standard deviation of 4.09. The stress score ranged from 0 to 21, with a mean of 4.76 and a standard deviation of 4.15.

**Table 2:** Scores of suicidal ideation, depression, anxiety, and stress.

Items	Range	Mean ± SD
Suicidal Ideation Scale overall scores	10-43	11 ± 4
Depression score	0-21	3.94 ± 4.28
Anxiety score	0-20	4.54 ± 4.09
Stress score	0-21	4.76 ± 4.15

Similarly, Table 3 shows the suicidal score in the demographic data using the independent t-test of significance to check for the independence between two mean values, and one-way ANOVA test was used to find if there is statistical significant difference between the means of three or more independent groups. ANOVA test was checked from the F-values in the statistics. Therefore, in this table, t-value of two independent mean values of marital status was significant, 2.6 (0.01), at the 0.05 level of significance. Similarly, mean value between two means in religion was also significant with a t-value of 2.2 (0.031). On the other hand, mean value in the group describing living conditions also has significant values of F = 5.4 (0.005) at the 0.05 level of significance. The rest of the results did not show any significance.

**Table 3:** Association between sociodemographic data and Suicidal Ideation Scale overall score. F, one-way ANOVA; t, independent samples t-test *p < 0.05, **p < 0.01 ANOVA, analysis of variance

Sociodemographic data		Suicidal Ideation Scale overall scores (mean ± SD)	Test (P)
Gender	Female	11.3 ± 4.0	t=0.05 (0.958)
	Male	11.3 ± 3.9	
Age	20-29 years	11.7 ± 5.7	F=2.1 (0.099)
	30-39 years	11.7 ± 4.5	
	40-49 years	10.7 ± 2.4	
	50 years and more	10.7 ± 1.9	
Nationality	Saudi	10.5 ± 1.2	t=1.1 (0.305)
	Non-Saudi	11.4 ± 4.1	
Marital status	Single	12.4 ± 6.3	t=2.6 (0.01)*
	Married	10.9 ± 2.3	
Religion	Muslim	10.7 ± 1.9	t=2.2 (0.031)*
	Non-Muslim	11.4 ± 4.3	
Living condition	With family	10.9 ± 2.8	F=5.4 (0.005)**
	With friends	10.9 ± 2.0	
	By myself	12.4 ± 6.4	
Department	Department of Emergency Medicine	11.1 ± 4.3	F=0.864 (0.567)
	Obstetrics and Gynecology	10.6 ± 2.9	
	Outpatient Department	12.2 ± 5.3	
	Surgery	11.5 ± 3.5	
	Pediatrics	11.0 ± 3.7	
	Operating rooms	11.4 ± 2.7	
	Medicine	12.5 ± 7.3	
	Critical care	11.2 ± 2.6	
	Ophthalmology	11.5 ± 4.0	
	Dental nurse	10.6 ± 1.9	
	Others	11.3 ± 4.0	
Years of experience	<5 years	11.1 ± 3.2	F=0.70 (0.551)
	5-10 years	11.7 ± 5.1	
	10-20 years	11.3 ± 3.8	
	More than 20 years	10.8 ± 2.7	

**Figure 1:**
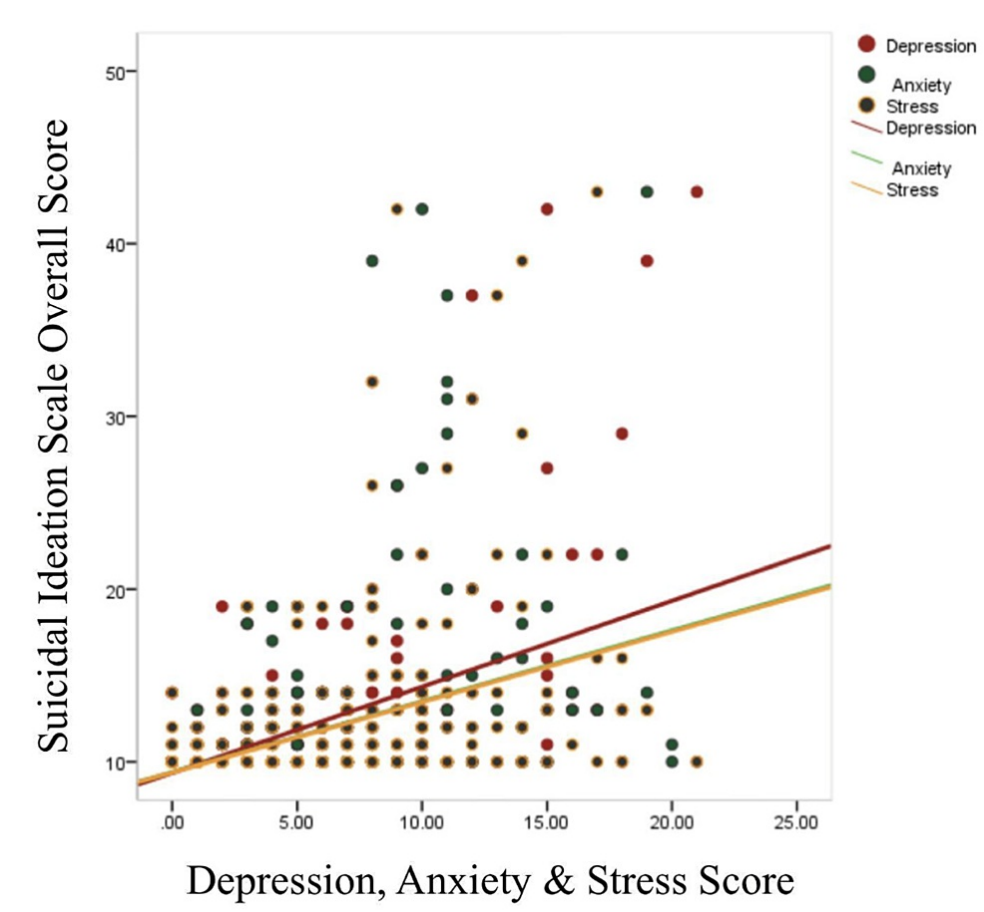
Correlation scatter diagram of Suicidal Ideation Scale scores with the Depression, Anxiety, and Stress Scale

In addition, in Table 4, the correlation between depression, anxiety, and stress scores with the help of Pearson correlation coefficient is also shown, where their correlation is compared with the Suicidal Ideation Scale. Therefore, correlation between depression score and Suicidal Ideation Scale is r=0.53 (0.001), anxiety score and Suicidal Ideation Scale is r=0.42 (0.001), and stress score and Suicidal Ideation Scale is r=0.42 (0.001). Thus, values indicate significant correlation values in all the three scales at 0.001 level of significance. Correlation is also demonstrated in the scatter plot diagram (Figure 1).

**Table 4:** Correlation of mental health and Suicidal Ideation Scale overall scores ^┼^Pearson correlation coefficient, *Correlation is significant at the 0.01 level (two-tailed).

Mental health	Suicidal Ideation Scale overall scores	
	r^┼^	P-value
Depression score	0.53^*^	0.001
Anxiety score	0.42^*^	0.001
Stress score	0.42^*^	0.001

Nurses who had a score above 10 were 103, whereas the remaining 316 nurses had a score of 10. Female and male nurses who had a score above 10 were 94 and 9, respectively, whereas the rest of female and male nurses who scored 10 were 287 and 29, respectively. Considering these criteria, the overall calculated prevalence of suicidal ideation among nurses in KSUMC was 24.58%. As for the prevalence of suicidal ideation among female and male nurses in KSUMC, it was 24.67% and 23.68%, respectively.

## Discussion

The study measured the prevalence of suicide ideation among nurses in KSUMC. It also investigated the primary causes and risk factors that could lead to an increase in suicidal ideation. We found being single, living alone, and being non-Muslim to be sociodemographic risk factors most associated with suicidal ideation. In addition, depression, anxiety, and stress were found to be significantly correlated to suicidal ideation, with depression being the most related to suicidal ideation. The study also found no significant difference in gender concerning suicidal ideation.

We hypothesized that more than 18.3% of working nurses are prone to suicidal ideation based on a study conducted in Hong Kong, and our results were consistent with our hypothesis, with a prevalence of 24.58% [25]. As for the risk factors, a study conducted in Brazil indicated that marital status is an important associated risk factor for developing suicidal ideation, and it also appeared to have an effect on the emergence of depression and stress [18]. Also, a vast number of studies support the correlation between marriage and improved health outcomes, as well as decreased suicide rates [19], which is consistent with our findings. Another major risk factor is the living conditions. A Chinese study indicated that living with a partner was statistically associated with an increased likelihood of suicidal ideation in nurses, and this contradicts our findings [21]. The study results were similar to a study conducted in Hong Kong, where religion is an important associated risk factor for suicidality [25]. Furthermore, a meta-analysis found that adults with a religious affiliation had a lower suicide risk [9]. Our gender correlation finding is supported by a study conducted in Canada that measured the suicidal ideation rates among male and female nurses and found no significance [20]. However, another study conducted in Hong Kong was inconsistently suggesting that female nurses are at higher suicide risk than male nurses [25].

Various risk factors have been associated with suicidal ideation in nurses, but the most crucial three were depression, anxiety, and symptoms of stress [25,27]. Many prospective studies have revealed that depression and hopelessness are associated with a higher risk of subsequent suicidal ideation, attempt, and death, with other studies indicating a 20- to 30-fold increase in risk, which concurs with this study [28]. As evidence of this, depression and hopelessness are consistently featured within the risk-factor guidelines developed by major national and international organizations and commonly integrated into structured suicide-risk assessments [28]. Additionally, the World Health Organization (WHO) listed depression as an antecedent of suicidal behavior [3]. On the other hand, a study that was conducted in Hong Kong revealed that there is no link between anxiety and suicidal ideation, which is inconsistent with our findings [25].

As far as we know, this is one of the few studies that focus on suicide ideation among nurses in the Kingdom of Saudi Arabia, and hopefully it would encourage other researchers in Saudi Arabia to conduct similar studies. Our findings could not be applied to other medical cities in Riyadh or other Saudi cities as the current study is limited due to the inclusion of nurses from only KSUMC. Another limitation is that a simple random sampling technique could not be conducted due to the lack of a list of the nursing staff that work in KSUMC to preserve confidentiality. Also, the study might have engaged participants with a similar experience since the respondents chose to participate in the survey, which might have resulted in overestimation and selection bias.

Finally, it is crucial to highlight the importance of such studies due to the scarcity of the data and publications in this domain. For this reason, we found this research to be a critical step in providing data that will enable the nursing department at KSUMC to develop solutions and promote awareness, especially since our study resulted in nearly a quarter of nurses having suicidal ideation. In terms of study setting, the occupational clinic at King Khalid University Hospital only opens once a week; however, these hours should be extended due to the hectic schedules that most nurses have, which may prevent them from receiving essential care. Another preventative measure that could be further implemented is the crisis lines as it is a standard component of a public health approach to suicide prevention and protection. A free, easily accessible confidential suicide prevention hotline needs to be established in Saudi Arabia to support those people who experience a suicidal crisis or emotional disorder. Future studies should examine the efficacy of these suggested preventative measures in more detail and determine the causality of suicidal ideation through conducting a qualitative semi-structured interview to understand more about what motivates and deters people from committing suicide.

## Conclusions

Suicide has many aspects that are closely correlated to the individual associated risk factors, work-related factors, and mental health. The aim of this study was to highlight the significance of the risks associated with developing suicidal ideation in nurses, which are often disregarded, even by nurses themselves. The results provided evidence that the respondents who were non-Muslims, living by themselves, and single are more prone to suicidal ideation and showed that there is no significant difference in relation to gender. The findings of our study also revealed that mental disorders such as depression, anxiety, and stress may lead to an increased risk of suicidality.

## Appendices

Appendix A 

**Table 5:** Depression, Anxiety and Stress Scale (DASS21) Please read each statement and choose 0,1,2 or 3 which indicates how much the statement applied to you over the past week. There are no right or wrong answers. Do not spend too much time on any statement. Please be aware that the rating scale is as follows: 0: Did not apply to me at all – NEVER. 1: Applied to me to some degree, or some of the time – SOMETIMES. 2: Applied to me to a considerable degree, or a good part of time - OFTEN. 3: Applied to me very much, or most of the time – ALMOST ALWAYS

Statement applied to you over the past week	0	1	2	3
I found it hard to wind down.				
I was aware of dryness of my mouth.				
I couldn’t seem to experience any positive feeling at all.				
I experienced breathing difficulty (e.g. excessively rapid breathing, breathlessness in the absence of physical exertion).				
I found it difficult to work up the initiative to do things.				
I tended to over-react to situations.				
I experienced trembling (e.g. in the hands).				
I felt that I was using a lot of nervous energy.				
I was worried about situations in which I might panic and make a fool of myself.				
I felt that I had nothing to look forward to.				
I found myself getting agitated.				
I found it difficult to relax.				
I felt down-hearted and blue.				
I was intolerant of anything that kept me from getting on with what I was doing.				
I felt I was close to panic.				
I was unable to become enthusiastic about anything.				
I felt I wasn’t worth much as a person.				
I felt that I was rather touchy.				
I was aware of the action of my heart in the absence of physical exertion (e.g. sense of heart rate increase, heart missing a beat).				
I felt scared without any good reason.				
I felt that life was meaningless.				

Appendix B 

**Table 6:** Suicidal Thoughts Assessment (SIS) This questionnaire consists of 10 statements. Please read each carefully and then choose the number for each item that best describes the way you have felt over the past week, including today. Be sure to choose only one number for each item. Please be aware that the rating scale is as follows: 1: Never 2: Infrequently 3: Sometimes 4: Frequently 5: Always

Statements describing the way you have felt over the past week	1	2	3	4	5
I have been thinking of ways to kill myself.					
I have told someone I want to kill myself.					
I believe my life will end in suicide.					
I have made attempts to kill myself.					
I feel life just is not worth living.					
Life is so bad I feel like giving up.					
I just wish my life would end.					
It would be better for everyone involved if I were to die.					
I feel there is no solution to my problems other than taking my own life.					
I have come close to taking my own life.					
